# Regulation of blood-screening *in vitro* diagnostics in sub-Saharan African countries remains a challenge

**DOI:** 10.3389/fmed.2023.1252721

**Published:** 2023-10-03

**Authors:** Goran Abdurrahman, Washington Samukange, Nyambe Lyoko, Negus Onai Shonhiwa, Chancelar Kafere, C. Micha Nübling, Rosanna W. Peeling, Jens Reinhardt

**Affiliations:** ^1^TÜV Rheinland LGA Products GmbH, Cologne, Germany; ^2^Paul-Ehrlich-Institut, Langen, Germany; ^3^Division of Pharmacoepidemiology and Clinical Pharmacology, Faculty of Science, Utrecht Institute for Pharmaceutical Sciences (UIPS), Utrecht University, Utrecht, Netherlands; ^4^Zambia Medicines Regulatory Authority, Lusaka, Zambia; ^5^Medicines Control Authority of Zimbabwe, Harare, Zimbabwe; ^6^London School of Hygiene and Tropical Medicine, London, United Kingdom

**Keywords:** IVD, regulation, blood transfusion, sub-Saharan Africa, BloodTrain

## Abstract

According to the World Health Organization, blood must be screened for major transmitted infections before transfusion to prevent the possibility of passing an infection to the recipient. For accurate detection of infectious disease pathogens in the blood of donors, *in-vitro* diagnostic medical devices (IVDs) of high specificity and sensitivity should be used. In mature healthcare systems, the regulatory authorities authorize the usage of devices with the highest performance capabilities, which are also controlled through active market oversight. However, in Sub-Saharan African countries, the regulation of IVDs is often poorly developed. With the lack of stringent regulatory oversight, IVDs of poor quality can be put on the market and used for blood donor screening, which, ultimately, poses a great public health threat. The BloodTrain is a humanitarian project from the Germany Federal Ministry of Health that aims to help strengthen the regulatory authorities in Sub-Saharan partner countries. Here, we present the status of IVD regulation in the partner countries and the objectives that the BloodTrain project aims to achieve in the region toward regulating IVDs.

## Introduction

Sub-Saharan Africa is home to 1.1 billion human beings (1) and is considered the world’s poorest but growing region (2). Although the healthy life expectancy has expanded in sub-Saharan Africa in the past decades (3), the region still faces major health challenges combined with a high prevalence of infectious diseases. Examples of the infectious diseases widespread in the region include HIV, HBV, HCV, Malaria, and syphilis (4–6). The agents of these infectious diseases are blood born and can be transmitted from one person to another through medical interventions, such as blood transfusions.

The availability of safe blood supply in some African countries has long been known to be challenging. This is due to systematic insufficiencies across multiple levels, including blood policy, regulatory procedures, funding, collections, testing, and post-transfusion surveillance. Interventions from the World Health Organization (WHO) and other foreign government bodies, together with an influx of external funding, greatly helped with the advancement in blood safety in sub-Saharan African countries in the early 2000s. External support has been associated with increased recruitment of voluntary blood donors and extended screening for major transfusion-transmitted infections (TTIs). In the meantime diminishing external support has posed a major challenge for regional transfusion services (7). According to the WHO, all blood donations should be screened for major TTIs (8) prior to use (8). These include screenings for human immunodeficiency virus (HIV), hepatitis B virus (HBV), hepatitis C virus (HCV), and syphilis. A major challenge in sub-Saharan African countries is that the rate of TTIs among blood donors is significantly high. For instance, in sub-Saharan African countries, the prevalence of TTIs in donated blood are as follows: HIV (0.06–4.13%), HBV (0.07–13.7%), HCV (0.005–3.8%), and syphilis (0.04–3.67%) (9). However, in developed countries, the rate of TTIs are significantly lower. As an example, in Germany the prevalence of the TTIs include HIV (less than 0.001%), HBV (0.004%), HCV (0.003%), and syphilis (0.003%) (9).

With challenges spanning the entire blood-safety chain, the sub-Saharan African region is still to improve the safety of blood and blood components. As highlighted by the WHO (8), one of such major elements is the founding of an appropriate regulatory system. In this context, regulation of the *in vitro* diagnostic medical devices (IVDs) is the first step of such a regulatory framework that assures the availability of robust and sensitive IVDs to deliver an accurate test result of potential infections during blood donations.

According to the International Medical Device Regulators Forum (IMDRF), an IVD is “a device, whether used alone or in combination, intended by the manufacturer for the *in vitro* examination of specimens derived from the human body solely or principally to provide information for diagnostic, monitoring, or compatibility purposes. This includes reagents, calibrators, control materials, specimen receptacles, software, and related instruments, or apparatus” (10).

IVDs should be regulated through legislation that describes the manufacturer’s obligations by referring to technical and clinical requirements. The regulatory authorities guide manufacturers through these technical requirements by providing documents on detailed technical standards that a manufacturer must follow before placing an IVD product on the market. Those documents are expected to provide the manufacturer with specifications, guidelines or characteristics, and acceptance criteria for the design and manufacturing of IVDs (11). Furthermore, guidance on how to design, execute and interpret performance evaluation studies is key. In developed nations, IVDs with potential false results associated with high risk for the individual or the public are regulated through stringent regulatory frameworks and guidelines that thoroughly assess the device before putting it on the market. However, in most African countries, IVD regulation seems to be poorly developed. A report by the (WHO) in 2017 has shed light on this issue and found that 40% of countries in the African region have no regulatory framework for medical devices, 32% have some regulations, and the remaining 28% have no available data (12).

In the lack of a stringent regulatory structure, IVDs of poor quality with low sensitivity and specificity can be put on the market and used to screen blood donations, which consequently poses a great risk of transmitting TTIs. Furthermore, due to the lack of local regulatory authorities, African countries often rely on CE-marked or FDA-approved devices without further assessments/evaluations. This may not fully help with the local situation in Africa, e. g. regarding the regional distribution of virus subtypes; those devices were initially developed and designed mainly for users in other geographical locations. Moreover, differences in patient populations, ethnicity, demography, and other socioeconomic factors, along with differences in pathogens and sub-type prevalence in different geographical locations, are proven to be crucial to be considered during IVD manufacturing.

The WHO performs evaluations on specific IVDs to determine whether the product meets the prequalification requirements for safety, quality, and performance, subjecting individual diagnostic tests through a standardized assessment procedure, called Diagnostics Prequalification (PQ) program. This program is especially important for sub-Saharan African countries, as the requirements are designed in a way to meet the needs of resource-limited settings. Through the PQ programs, the WHO aims to guide interested United Nations agencies and the WHO Member States without mature regulatory assessment systems for IVDs. For an IVD to be eligible for assessment through this program, the device in question should fulfill a global need and be suitable for use in resource-limited settings (13). Although the WHO PQ program fills some of the regulatory gaps in the sub-Saharan African counties, its portfolio does not focus especially on the blood screening IVDs, which is beyond the program’s capacity. Therefore, stringent IVD regulation in Africa is still required.

## The WHO’s recommendations for an optimal regulatory structure

In 2014, WHO adopted a resolution that sets out the framework for strengthening regulatory systems focusing on medical devices and IVDs. The resolution states that “effective regulatory systems are an essential component of health system strengthening and contribute to better public health outcomes.” It also emphasizes that “inefficient regulatory systems can be a barrier to access to safe, effective, and quality medical products” (14). In light of this resolution, the globally growing interest in IVDs, and the lack of proper regulatory structures, the WHO developed a global regulatory model that recommends guiding principles, harmonized definitions, and specifies the qualities of operative and efficient regulation to be accommodated within the binding and enforceable legislation (12). This model is mainly built on the guidance documents developed by the IMDRF and its predecessor, the Global Harmonization Task Force (GHTF). The model is written for the governmental regulatory branches that develop and establish a system for medical device regulations legislation. This model also contains information on how a manufacturer must demonstrate to a regulatory authority that its medical device has been developed to be safe and performs as intended. The model also summarizes the functions of a regulatory entity and essential principles for regulation. It also presents a stepwise approach to implementing and enforcing regulatory controls of IVDs from the basic to the advanced level.

This model expects the National Regulatory Authorities (NRAs) to set out regulatory requirements for the entire process of putting an IVD into service, including premarket activities, placing on the market, and postmarket activities. IVD regulation must have a sound basis in law that identifies entities subject to regulation. The NRAs that are at the beginning of setting up a regulation, the model sets out a path and requirements to build a basic-level regulation that can be a foundation of a more mature and “expanded-level” regulation. According to this model, a basic-level regulatory foundation is effective oversight of the IVDs placed on the market. This is achieved through laws obliging IVD manufacturers or their authorized representatives to register at the NRAs. And also to control imports through approval of importation documents before shipment and verification of imported products.

Once the basic-level controls have been implemented, NRAs can consider implementing more advanced controls in different aspects of the regulation (premarket, placing, and postmarket) based on the priority of the country. Important features of NRAs functioning at the expanded level of regulation are to create oversight of the clinical investigation, appoint conformity assessment authorities, and recognize standards. Moreover, performing the review of submissions for compliance with the essential principles and more robust postmarket surveillance and vigilance reporting are expected to be covered by the NRA.

In order to assess a regulatory authority’s status, the WHO has also developed the Global Benchmarking Tool (GBT) plus medical devices. The benchmarking activities within the WHO started in 1997; it has been updated and expanded over the years, with the newest requirements for global benchmarking and assessment of the regulation of medical devices (GBT plus medical devices) published in 2022. Another version of the WHO GBT, the GBT plus blood, defines blood regulation and includes requirements of medical devices used for blood, including blood screening IVDs. The GBT enables the NRAs to either self-evaluate their own regulatory authorities or be independently assessed and in the process pinpoints good regulatory practices and areas for improvement (15). The GBT integrates the concept of “maturity level” or ML (adapted from ISO 9004), allowing regulatory authorities to be evaluated based on the overall “maturity” of the regulatory system on a scale of 1 (existence of some elements of the regulatory system) to 4 (operating at an advanced level of performance and continuous improvement) (16).

Recently, the WHO has established the WHO Listed Authority (WLA) in an attempt to globally recognize regulatory authorities functioning at a high level of performance. The WLA framework’s application aims to promote access to and supply medical products of high quality, which are safe and effective. The framework offers the optimal use of limited resources by enabling trust in the work products and decisions of reliable agencies in the decision-making of regulatory authorities. This ultimately fosters regulatory convergence, harmonization of approaches, and international collaboration, consequently improving good regulatory practices (17).

On the basis of experience with procedures for prequalified medicines and vaccines, WHO recently developed a Collaborative Registration Procedure (CRP) for IVDs. The procedure aims to facilitate and expedite national marketing authorization processes and post-registration regulatory life-cycles of prequalified IVDs by enabling NRAs to make use of outcomes of WHO PQ assessments through confidential information sharing (18). With access to all information and reports from WHO PQ, NRAs make their own independent decisions in line with their legislative and other applicable national regulatory requirements.

Globally, many countries still lack the foundation of an efficient and reliable regulatory structure. According to the WHO, among its 194 Member States, only 26% (50 countries) have mature regulatory agencies (maturity level 3 and above). In contrast, the remaining 74% (144 countries) have suboptimal regulatory systems, with over half (99 countries) at the lowest level of maturity (15). In sub-Saharan Africa, three countries are at Maturity Level 3 for medicines, namely Ghana, Nigeria, and Tanzania and two countries are at Maturity level 3 for vaccines, namely Egypt and South Africa, according to the WHO list from November 2022.

## BloodTrain

BloodTrain is a program within the Global Health Protection Program (GHPP), established in 2016 under the umbrella of the Federal Ministry of Health of Germany. BloodTrain supports several African partner countries in capacity-building developments to improve patient access to safe blood and blood components. A key element in this context is a functioning regulatory structure that can assure blood and blood products’ availability, safety, and quality. The BloodTrain team comprises several international scientists spanning the fields of virology, microbiology, and immunology who have been trained and experienced in regulatory aspects of blood and blood products. The BloodTrain team works closely with sub-Saharan African partner countries to analyze the local conditions in African countries, take appropriate measures in dialog with local authorities and blood transfusion services, and establish comprehensive and structured training for colleagues from the African partner countries. The partner countries include Zambia, Zimbabwe, Ghana, Tanzania, and Nigeria. The aims and objectives of the BloodTrain program involve the following. First, the overall goal is to contribute to the improvement of the availability, safety, and quality of blood and blood products in sub-Saharan African partner countries. One aspect of achieving this is the availability of reliable blood-screening methods, which are guaranteed through the use of safe and effective IVDs. Therefore, the BloodTrain program is working toward strengthening the regulation status of blood screening IVDs in order to ensure reliable blood testing. Lastly, the long-term goal of BloodTrain is to establish a harmonized regulatory system for medicines throughout Africa in cooperation with New Partnership for Africa’s Development (NEPAD).

## BloodTrain’s approach to strengthening the regulation of IVDs

The BloodTrain program is adopting a blended-learning approach, combining both online and face-to-face training. This approach is particularly important as during the COVID-19 pandemic and travel restrictions, through using the e-Learning tool (Moodle), the BloodTrain continued to deliver workshops, training, and meetings with regulators from NRAs of African partner countries. These online teachings are complemented by onsite workshops, in which BloodTrain trainers meet with regulators face-face and conduct training, covering different regulatory aspects of IVDs.

From February to August 2018, the BloodTrain used the WHO GBT plus blood to assess the status of implementation of regulation for blood-screening *in-vitro* diagnostics in 10 countries in Africa. This allowed the BloodTrain team the opportunity to determine the types of capacity building support activities necessary to build capacities for regulation of blood-screening *in-vitro* diagnostic medical devices in NRAs in Africa. A placement for eight NRA staff from 4 countries in (Nigeria, Tanzania, Zambia and Zimbabwe) was organized at the Paul-Ehrlich-Institut in March 2019 to facilitate NRAs to understand the role Paul-Ehrlich-Institut (PEI) plays in supporting the assurance of quality and safety for blood-screening *in-vitro* diagnostics in Europe.

In September 2021, BloodTrain organized an online workshop on the assessment of the IVD technical file. BloodTrain gathered IVD experts from PEI, WHO, academics, and consultants to share their knowledge on IVD regulation in a 4-day workshop (19). Over 100 regulators of medical devices and IVDs from NRAs of 12 African countries joined this online workshop. These countries included Ethiopia, Ghana, Kenya, Liberia, Malawi, Nigeria, Rwanda, South Africa, Tanzania, Uganda, Zambia, and Zimbabwe. Five of these countries (Ghana, Nigeria, Tanzania, Zambia, and Zimbabwe) are BloodTrain partner countries, while the rest are countries where benchmarking of blood regulatory systems was previously conducted by BloodTrain.

In this workshop, the regulators were trained on how to assess technical files for blood screening IVDs. Participants were tutored on aspects related to the regulation of IVD medical devices and regulatory harmonization and reliance schemes for IVDs. This four-day workshop presented a unique opportunity and a great starting point for most participating NRAs to set up and/or strengthen their regulatory structures for IVDs.

**Figure 1:**
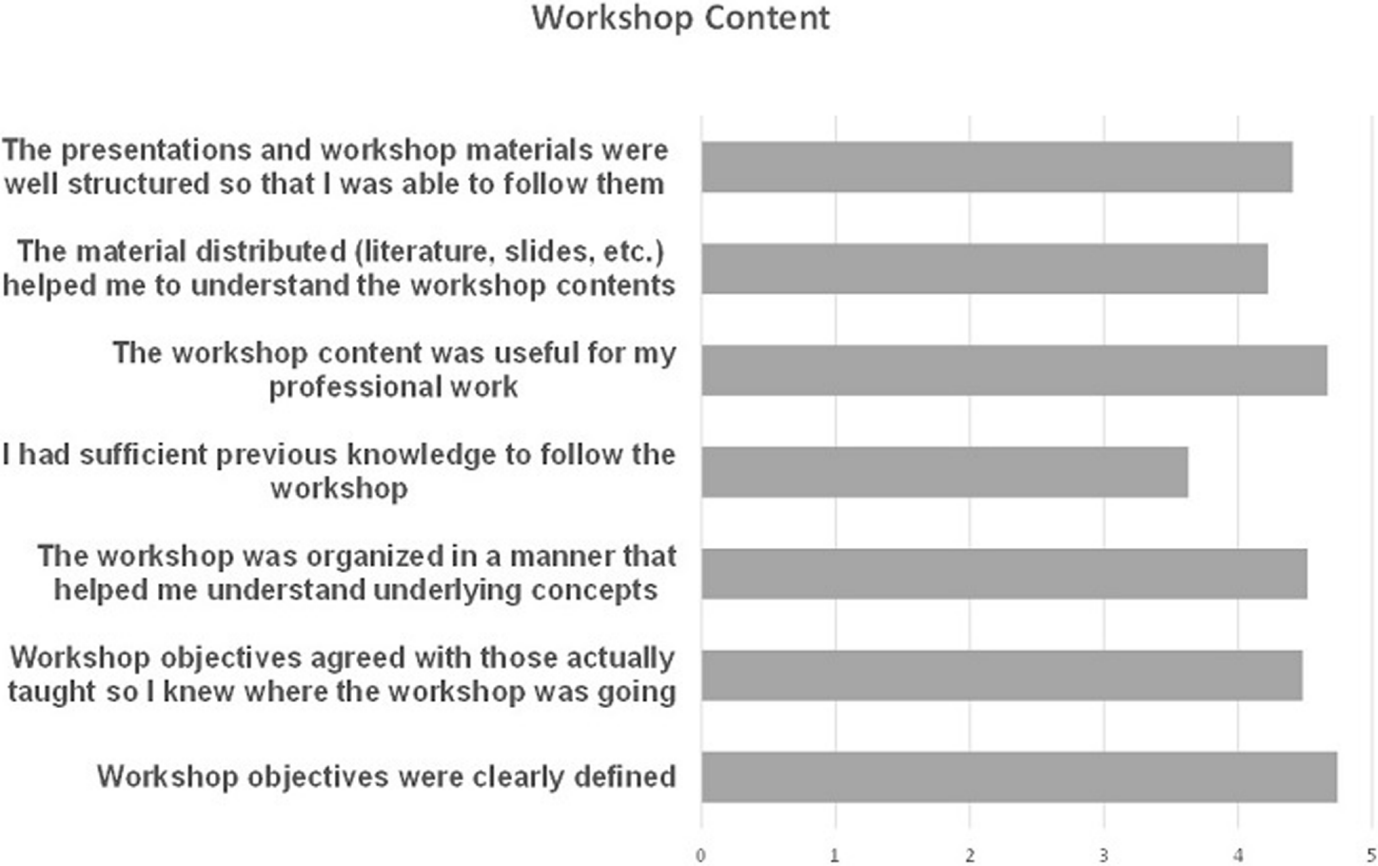
Results of the online survey performed after the online IVD workshop 2021. The results show a high overall satisfaction with the workshop content.

BloodTrain subsequently performed a survey among the participants of the workshop that was answered by 29 of the 107 participants (i.e., 27%). For the questions on the workshop content, the range of answer was from 1 (not at all satisfied) to 5 (totally satisfied). The overall response was highly positive (see Figure 1) and showed that there is a broad interest in the area of IVD regugulation in the participating African countries. According to the comments given in the survey, people showed a high interest in additional trainings and also suggested additional hands-on training with practical case studies. It was also commented that on site meetings would be more beneficial, as several participants experienced problems with the connectivity.

Following this online workshop, BloodTrain organized an onsite workshop in May 2022 that was only available for employees at the NRAs of Zambia and Zimbabwe. In this workshop, experts from BloodTrain and PEI trained the participants on how to critically assess a technical file of an IVD device submitted to their authorities. Participants were informed on all the components of a dossier, with a major focus on how to evaluate the different information presented in an IVD technical file or dossier. In this regard, participants were provided with mock dossiers of different fictitious IVDs. They were guided in evaluating the different information, such as stability studies, labeling and instructions for use, and performance studies. Lastly, experts from PEI informed the participants about the activities that PEI performs in the vigilance and testing of IVDs. Discussions were carried out on transferring and adapting PEI knowledge to the African partner countries.

## The status of IVD regulation in partner countries

In a second part of the meeting, the current status of IVD regulation in Zambia and Zimbabwe was presented by members of the respective agencies.

The Zambia Medicines Regulatory Authority (ZAMRA) is the national medicines regulatory body of Zambia, which is responsible for regulating IVDs in the country. In 2020, the ZAMRA implemented a regulatory guideline concerning marketing authorization, performance evaluation, and import controls on a selected scope of IVDs. Using a risk-based approach, the scope of the IVD regulation in Zambia covers the blood diseases of importance to transfusion medicine, such as HIV and syphilis. However, ZAMRA is currently suffering from efficiently and effectively executing its mandate in regulating IVDs in the country mainly due to the challenges that ZAMRA is currently facing in terms of shortage of human resources and limited capacity building but also engagement with industry and regulatory compliance.

In Zimbabwe, the Medicines Control Authority of Zimbabwe (MCAZ) is a national authority for medical products regulation. It derives its legal mandate from its establishing act, the Medicines and Allied Substances Control Act (MASCA) [Chapter 15.03] (20). IVDs are currently not regulated in the country due to a lack of specific regulations and approved guidelines. However, quality control activities are undertaken for specific diagnostics for diseases such as Malaria. Activities such as batch testing and post-shipment testing of IVDs are being performed by the country’s National Microbiology Reference Laboratory (NMRL). Imported IVDs must also have traceable documentation for the batch-to batch-quality before they are approved for use in the country. MCAZ is currently in the process of drafting regulations for IVDs together with the NMRL and the Medical Laboratory & Clinical Scientists Council of Zimbabwe.

The status of the IVD regulation in the other partner countries was summarized by the BloodTrain.

In Ghana, the Food and Drugs Authority is the national authority for medicines and medical devices. The legal mandate to regulate medical devices is drawn from the Public Health Act 2012, Act 851 (21) where medical devices are defined and the regulatory controls implemented are also elaborated. The FDA Ghana has published three guidelines to support medical device regulation, namely the registration of medical devices, including diagnostics, registration of software as a medical device, and the guidelines for emergency use authorization. High-risk IVDs are classified according to the IMDRF risk classes, and all blood-related IVDs are regulated as either moderate-high or high-risk. These require prior registration or market authorization by the FDA before they are used in Ghana. A full technical file dossier is required to be submitted for evaluation and assessment.

In Tanzania, the Tanzania Medicines and Medical Devices Authority (TMDA) is responsible for regulating medicines, medical devices, diagnostics, biocides, and tobacco products. This mandate is drawn from the Tanzania Medicines and Medical Devices Act, Cap 219. Regulatory controls for medical devices, including IVDs, are laid down in the Tanzania Medicines and Medical Devices (Control of Medical Devices) Regulations of 2015. To facilitate the regulation of IVDs, TMDA developed and published specific guidelines, the Guidelines on Submission of Documentation for Registration of *In-Vitro* Diagnostic Devices (22). According to this guideline, registration of the IVDs at TMDA requires the submission of a detailed technical dossier, allowing the TMDA regulators the assessment of data to ascertain the quality, safety, and performance of the device. Using a risk-based approach, the guideline classifies blood screening IVDs as the highest risk class (class D).

In Nigeria, National Agency for Food and Drug Administration and Control (NAFDAC) is responsible for regulating medical devices, including IVDs. The NAFDAC’s Act Cap N1 Laws of the Federation of Nigeria 2004 (23) regulates and controls the manufacture, importation, exportation, distribution, advertisement, sale, and use of, among others, IVDs. NAFDAC reviews IVD technical dossiers before allowing the product to be used in the country.

## The future perspective

The ultimate aim of the BloodTrain program is to contribute to the availability of safe blood in sub-Saharan African countries by building well-functioning regulatory structures. The program is now in its 7th year and has achieved several milestones including benchmarking of 10 NRAs in Africa as well as online and onsite workshops on IVD regulation for African partner countries. On the topic of IVD regulation, BloodTrain is planning to continue working with the partner countries throughout the journey of drafting and implementing regulation at their corresponding NRAs. In doing so, BloodTrain continues to contribute to the capacity building at the regulatory authorities through conducting further workshops and training using its blended approach. A particular focus at the moment is to train the regulators on how to critically review IVD technical files and how to assess whether the IVD functions as intended and meets the safety and performance requirements. Next, BloodTrain, together with other experts at PEI, is helping the NRAs in reviewing and drafting their legislation on the IVD regulation. In order to do so, task teams will be created that will focus on creating and drafting those documents. Lastly, for the efforts put into this project to be maintainable and function in the long run, BoodTrain is working closely with NEPAD on its sustainability plans. The plan includes designating one of the partner countries as Regional Centres of Regulatory Excellence (RCOREs) for IVD regulation. An RCORE is an institution or partnership of institutions with specific regulatory know-how and established capacity in training and/or delivery of services in a particular regulatory function (24). The main aim of the designated RCOREs here is to support a regulatory workforce that enhances human and institutional capacity and contributes to improved IVD regulation in Africa.

At continental level, the AUDA-NEPAD through the AMRH program established the Africa Medical Device Forum (AMDF), a technical working group on medical devices including IVDs. Its core purpose is to establish a harmonized framework for medical devices regulation in line with the WHO Medical Devices Regulatory Framework Model. To this end, AMDF is supporting NRAs in Africa through development of continental guidelines for adaption or adoption by respective NRAs and technical capacity building activities such as special trainings on medical device regulation among other strategies. In the long-run, AMDF intends to coordinate and facilitate joint regulatory activities among NRAs for functions such as inspections, technical file assessment and post market surveillance of medical devices including IVDs.

Establishment of the African Medicines Agency (AMA) will transform regulation of medicines including IVDs in Africa. The AMA treaty entered into force in November 2021 paving way for the currently ongoing setting up of the agency’s operational structures (25). As a specialized agency of the African Union, AMA aims to enhance regulatory harmonization initiatives, optimize resource utilization through promoting cooperation and mutual recognition of regulatory decisions (26, 27). Furthermore, the agency will provide technical assistance and guidance on regulatory issues. AMA will work through pooling together expertise and capacities in IVD regulation across Africa through AMA will improve regulatory control and hence access to quality, safe and effective IVDs.

The CRP for IVDs will go a long way in promoting regulatory harmonization and capacity building in African NRAs as they will be learning from work performed by WHO experts in the process. For the collaborative procedure to work, there is need for collaboration among NRAs, WHO PQ and manufacturers of prequalified IVDs. To realize the full potential benefits of the procedure and improve timely access to safe and effective IVDs in Africa, there is need for continued awareness campaigns to sensitize both NRAs and manufacturers in order for them to capitalize through consistent application of the CRP. Going forward, wide application of the CRP for IVDs will improve accessibility to safe and effective diagnostics in Africa and other resource limited settings.

## Data availability statement

The original contributions presented in the study are included in the article/supplementary material, further inquiries can be directed to the corresponding author.

## Author contributions

GA, WS, NL, NS, CK, CN, RP, and JR have participated in concentualising, writing, and editing of this manuscript and fulfill the requirement of co-authorship. All authors contributed to the article and approved the submitted version.
